# Supplementary material for: Imaging the electron charge density in monolayer MoS2 at the Ångstrom scale
Source: Nat Commun. 2023 Jul 20;14:4363. doi: 10.1038/s41467-023-39304-9 (PMC10359339; doi:10.1038/s41467-023-39304-9)
Supplement: Supplementary file 1 — Supplementary Information [file 41467_2023_39304_MOESM1_ESM.pdf]

Supplementary Information: Imaging the electron charge density in monolayer MoS_2 at the Ångstrom scale

Joel Martis[1*], Sandhya Susarla[2,3,4*], Archith Rayabharam[5], Cong Su[6,7,8], Timothy Paule[6,7,8], Philipp Pelz[2,9], Cassandra Huff[10], Xintong Xu[1], Hao-Kun Li[1], Marc Jaikissoon[10], Victoria Chen[10], Eric Pop[10], Krishna Saraswat[10], Alex Zettl[6,7,8], Narayana R. Aluru[11], Ramamoorthy Ramesh[6,7], Peter Ercius[2†], Arun Majumdar[1‡]

[1]*Department of Mechanical Engineering, Stanford University, USA*

[2]*The National Center for Electron Microscopy (NCEM), The Molecular Foundry, Lawrence Berkeley National Laboratory, USA*

[3]*Materials Science Division, Lawrence Berkeley National Laboratory, USA*

[4]*School for Engineering of Matter, Transport and Energy, Arizona State University, USA*

[5]*Department of Mechanical Engineering, University of Illinois Urbana-Champaign, USA*

[6]*Department of Physics, University of California Berkeley, USA*

[7]*Department of Materials Science and Engineering, University of California Berkeley, USA*

[8]*Kavli Energy NanoScience Institute, University of California Berkeley, USA*

[9]*Institute of Micro- and Nanostructure Research & Center for Nanoanalysis and Electron Microscopy (CENEM), Department of Materials Science, Friedrich-Alexander-Universität Erlangen-Nürnberg (FAU), Erlangen, Germany*

[10]*Department of Electrical Engineering, Stanford University, USA*

[11]*Department of Mechanical Engineering, The University of Texas at Austin, USA*

---

[*] These authors contributed equally to this work
[†] corresponding author (email: percius@lbl.gov)
[‡] corresponding author (email: amajumdar@stanford.edu)

Supplementary Note 1

The simultaneously acquired ADF-STEM image was used to derive the nuclear charge image (Figure S5). The experimental ADF-STEM images are shown in Fig S5(a) and (b). The Mo and S were differentiated based on their relative intensity (Mo / S ratio of ~2.5) (Fig. S5(d)), corresponding to a scattering proportional to ~$Z^{1.7}$, where Z is the atomic number. To validate the experimentally observed ADF-STEM intensity, we performed multislice simulations using abTEM with probe aberrations and ADF detector inner/outer angles of 80 mrad and 200 mrad (Fig S5(c)), approximating the conditions used in the experiment. This confirms that the observed intensities are in line with those expected from Mo/S scattering.

We then assigned nuclear charges (42e- for Mo and 16e- for S) as delta functions at each atomic site (Fig S5(e)). Finally, this was convolved with the shape of an 80kV 30 mrad probe with empirically derived aberrations and a 0.7 Å FWHM Gaussian (to approximate the source size) to obtain the nuclear charge density image (Fig S5(f)).

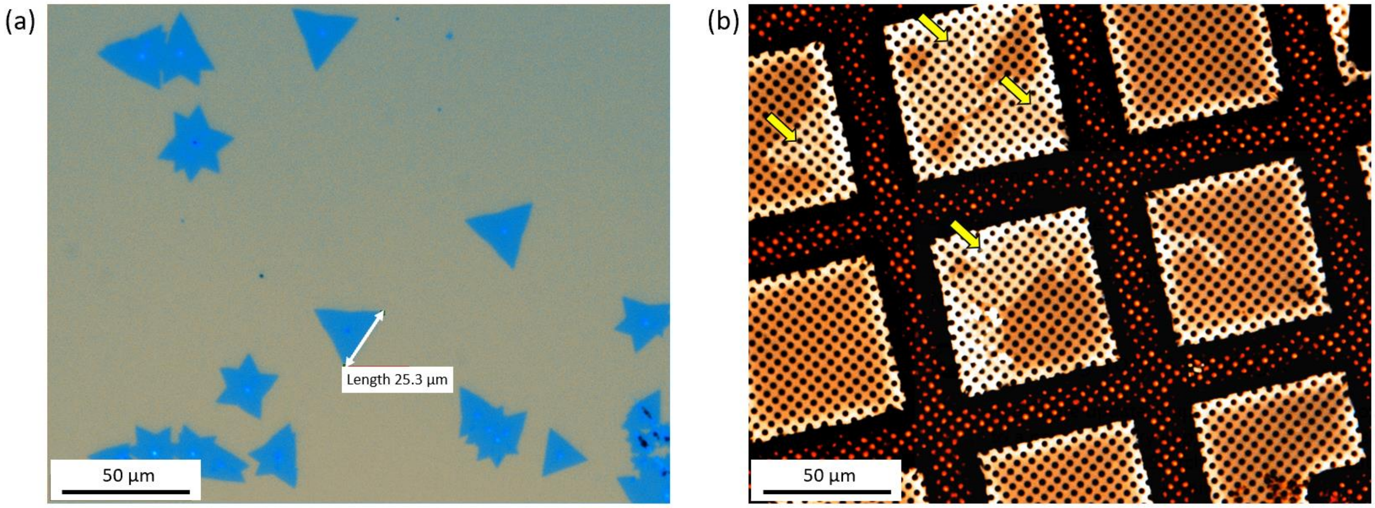

Supplementary Figure 1 | Optical images of MoS2 flakes (a) Optical image of MoS2 monolayer flakes on a SiO_2(285nm)/Si substrate (b) after transferring to a holey carbon TEM grid (yellow arrows indicate MoS2 flakes).

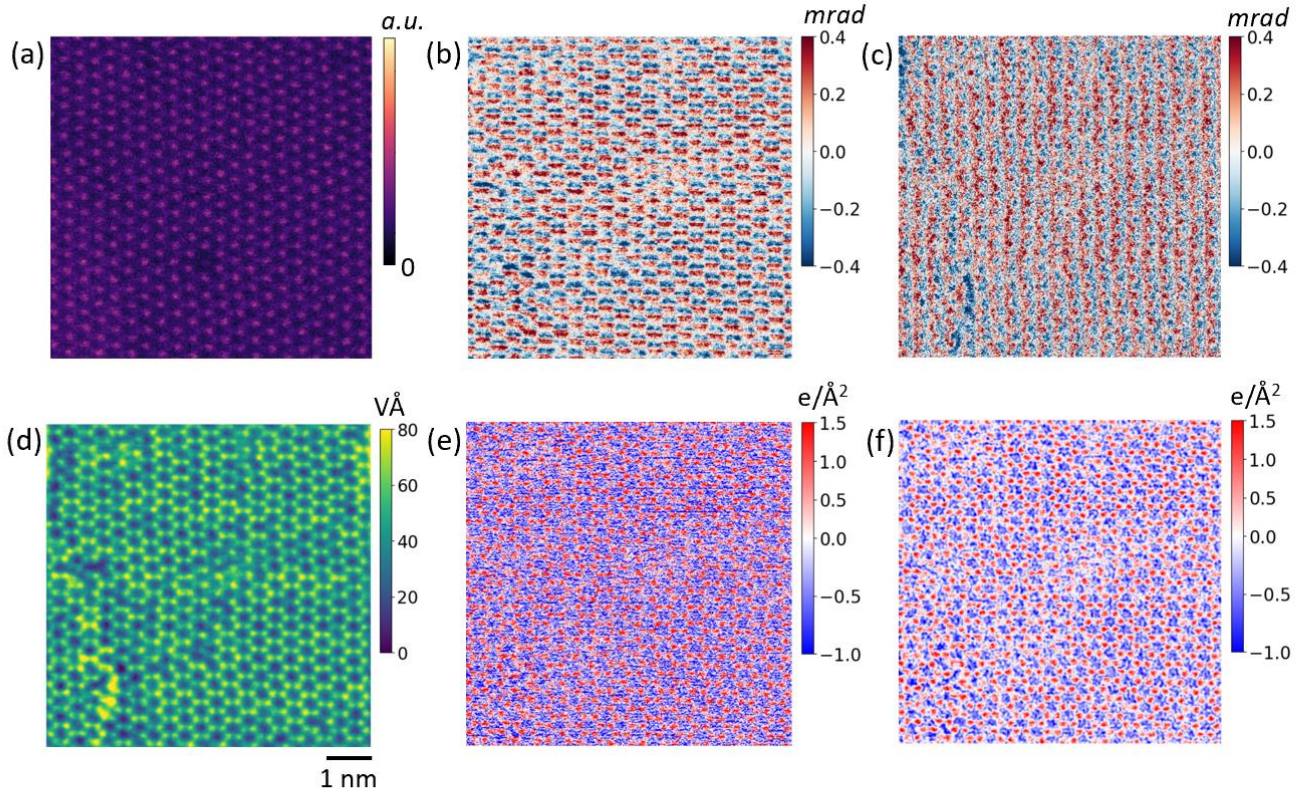

Supplementary Figure 2 | 4D-STEM dataset. (a) Simultaneously acquired ADF-STEM image, (b) & (c) CoMy and CoMx images, (d) electrostatic (projected) potential, (e) charge density, (f) 0.4 Ångstrom FWHM Gaussian blurred (e) to reduce high frequency noise.

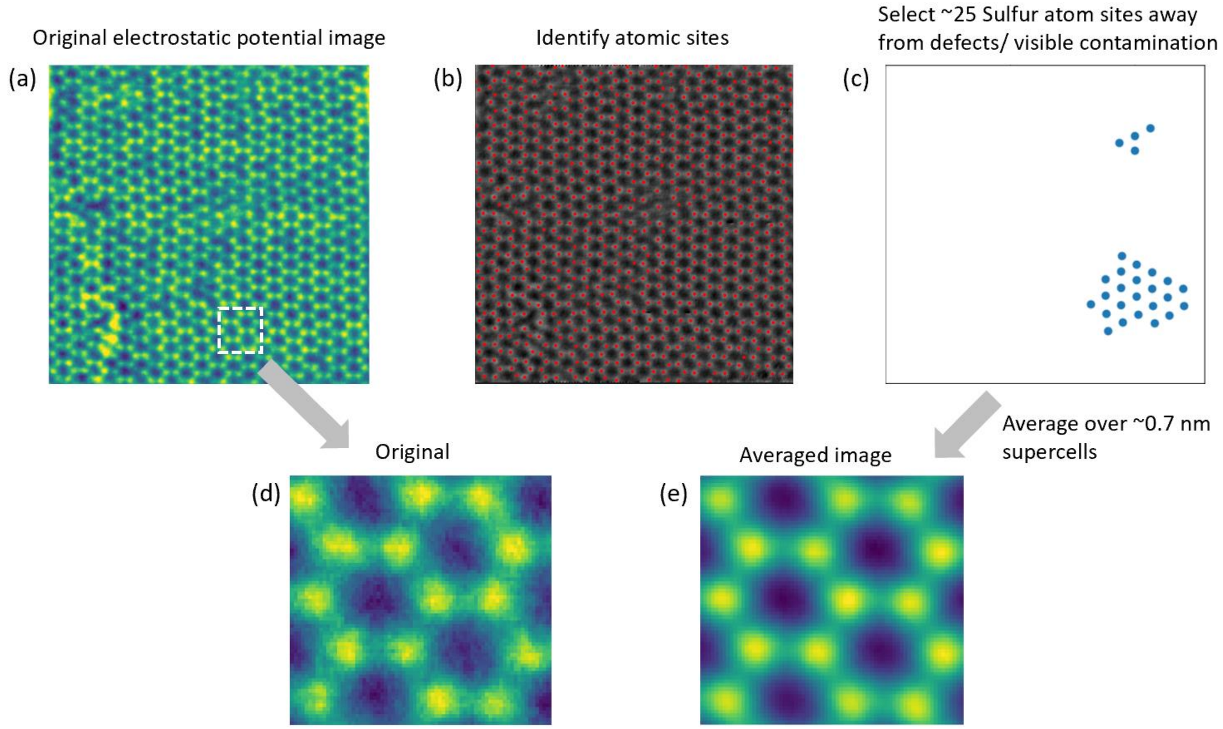

Supplementary Figure 3 | Unit cell averaging to improve SNR. (a) Original electrostatic potential image. (b) Greyscale version of (a) with atom positions (red dots) identified using AtomSegNet. (c) ~25 S atom positions away from defect sites and contaminants. (d) A region of (a) compared to the (e) averaged super cell image showing a significant improvement in SNR.

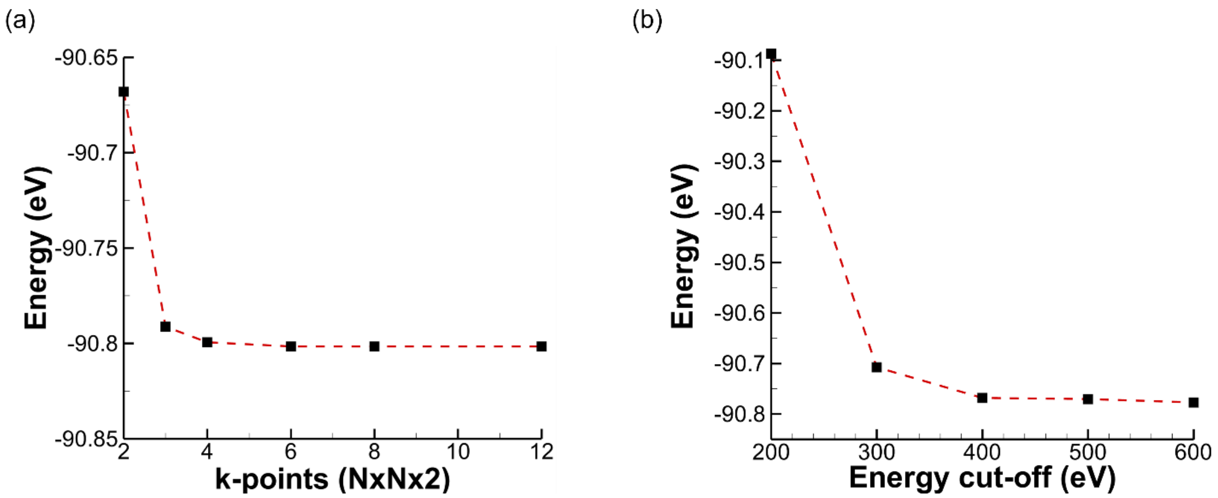

Supplementary Figure 4 | Convergence of DFT simulation. (a) The energy change with the number of k-points. (b) The energy change with the cut-off energy. A k-point grid of 8 × 8 × 2 and a cut-off energy of 500 eV are chosen to ensure proper convergence of the simulations and have good computational efficiency.

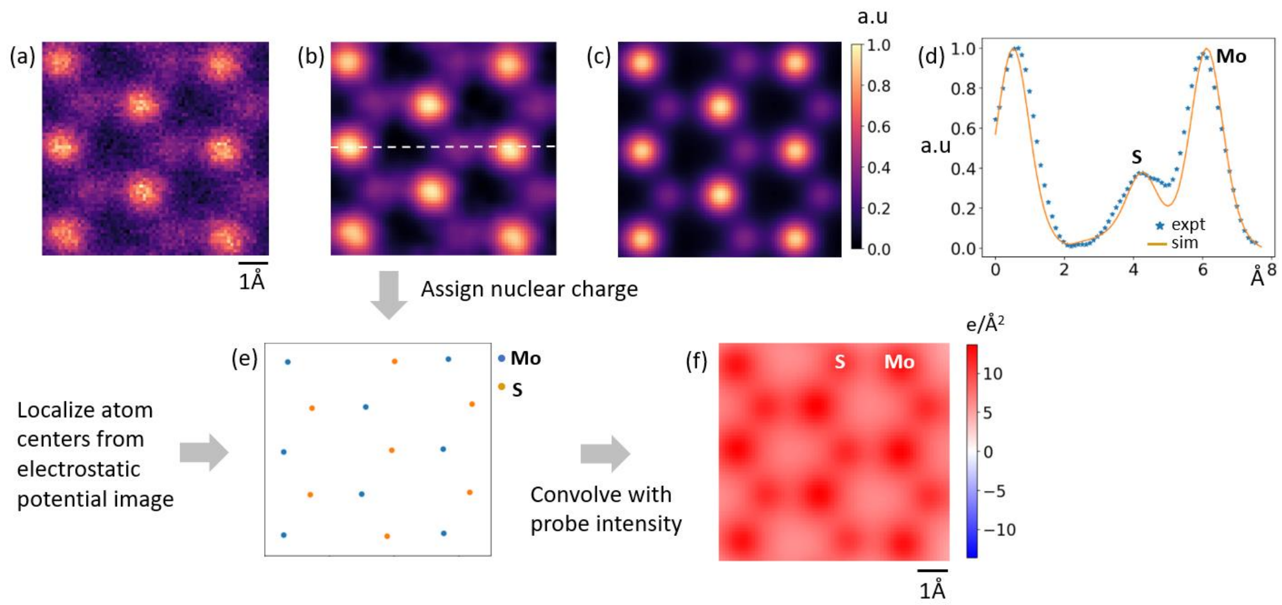

Supplementary Figure 5 | Determining the nuclear charge density image. (a) Averaged super cell ADF-STEM image. (b) Gaussian filtered version of (a) with a 0.4Å FWHM Gaussian. (c) Simulated ADF-STEM image (by the multislice method) using abTEM. (d) Line profiles comparing images (b) and (c). (e) Atom centers determined from the electrostatic potential image. Each position is assigned a known nuclear charge (Mo or S-S) determined from the ADF-STEM intensity. (f) Nuclear charge density obtained by convolving (e) with the aberrated probe intensity followed by source size blurring (0.7 Å FWHM Gaussian).

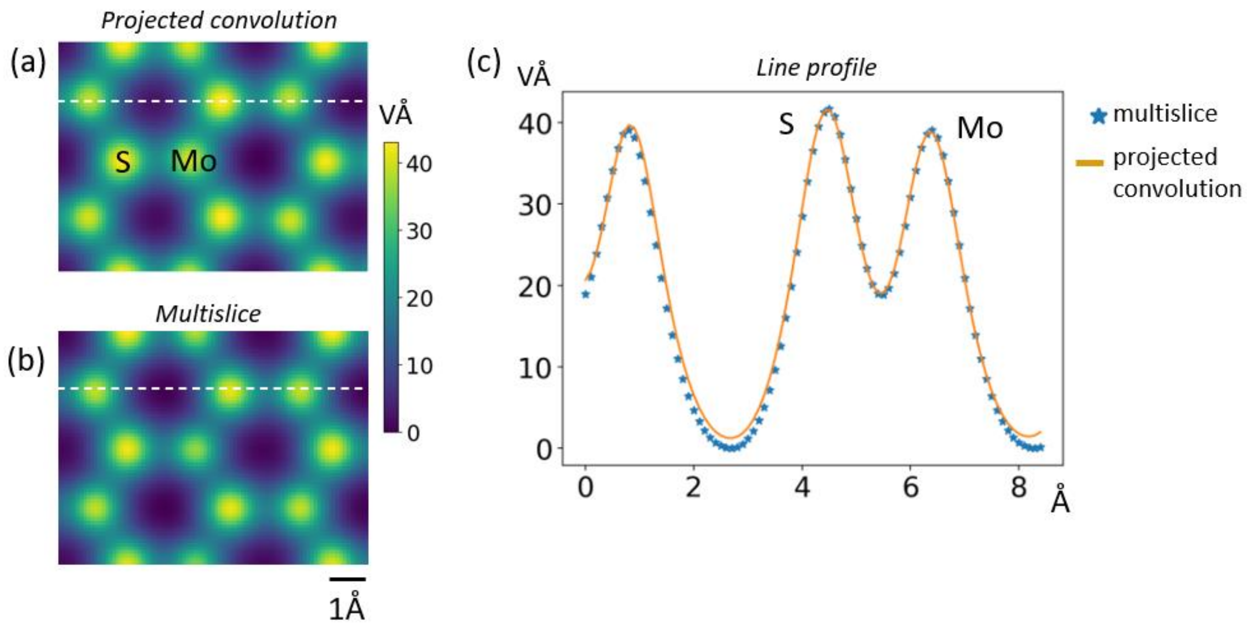

Supplementary Figure 6 | Comparing probe convolution (single slice) v/s multislice simulations. (a) Projected electrostatic potential convolved with a 30 mrad probe at 80 keV with 7.5 nm focal spread. (b) Multislice simulations with the same probe. (c) Line profiles across two Mo and one S site showing no difference in the two methods. The multislice simulations were carried out using the independent atom model in abTEM and included 5 frozen phonons.
